# Kidneys in the crossfire: insights into renal sarcoidosis

**DOI:** 10.1186/s12882-026-05057-y

**Published:** 2026-06-01

**Authors:** Zainah Sindhoo, Mohammed Askari Meghji, Mohamed Elewa, Nazar Mohammed, Geetanjali Gupta, Ismail Mohammed, Anand Vardhan, Durga A. K. Kanigicherla

**Affiliations:** 1 Renal Medicine, Manchester University NHS Foundation Trust, Manchester Academy of Health Sciences Centre, Manchester, UK; 2 Division of Cardiovascular Sciences, University of Manchester, Manchester, UK; 3 Department of Medicine, Ain Shams University, Cairo, Egypt

**Keywords:** Chronic kidney disease, End-stage renal disease, Granulomatous interstitial nephritis, Sarcoidosis

## Abstract

**Background:**

Renal Sarcoidosis is a rare disease and poorly described. We aimed to characterise the clinical presentation, histopathology, treatment response, and renal outcomes in patients with biopsy proven renal sarcoidosis.

**Methods:**

A retrospective observational study with biopsy-proven kidney disease in sarcoidosis, assessing demographics, clinical presentation, histology, treatments, and outcomes. Progression to CKD stage 4/5 or requiring dialysis was the primary outcome. Group comparisons and Cox proportional hazards models were used to identify predictors of the primary outcome.

**Results:**

Of the 32 patients, 44% presented with kidney disease. In others, it was noted 2.5 years after diagnosis of extra-renal sarcoidosis. Granulomatous interstitial nephritis was the most common histology seen (59.4%). Steroids were used in 78.1%, predominantly prednisolone; 56% received high-dose regimen (30 mg/day or more). Over a follow up of 6 years, 9 patients experienced CKD 4/5. Patients who developed primary outcome had significantly lower eGFR at the time of presentation to nephrologist (median 20 vs. 43 mL/min, *p* = 0.049), and in univariate Cox analysis, younger age at diagnosis was associated with primary outcome (HR 0.95, 95% CI 0.91–1.00; *p* = 0.039). During follow up, 10 (31%) patients developed relapse, 4 (12.5%) patients progressed to ESKD, and 3 (9%) patients died.

**Conclusion:**

Renal sarcoidosis is associated with incomplete renal recovery and carries a substantial risk of progression to advanced CKD or ESKD. Younger age and severe renal impairment at presentation were associated with poor renal outcomes, although these findings should be interpreted with caution. Our findings underscore the need for early diagnosis, structured follow-up, and multicentre prospective registries to refine prognostic tools and guide long-term care.

**Clinical trial number:**

Not applicable.

## Background

Sarcoidosis is a multisystem inflammatory disease characterised by the formation of non-caseating granulomas [1] and presents with protean manifestations. The most affected parts of the body include the lungs, musculoskeletal system, eyes, skin, liver and kidney. Whilst pulmonary sarcoid has been well documented and extensively studied [2], there is a notable lack of research on sarcoidosis as a cause of kidney disease and how renal sarcoid is managed.

The incidence of sarcoidosis ranges from 5 to 64 per 100,000 population, with a female predominance and peak onset between ages 25 and 60 [3, 4]. Higher rates are reported among African American women [4, 5]. Comorbidities often include pulmonary and cardiovascular disease, and multisystem involvement is associated with increased morbidity and mortality [6].

The aetiology is multifactorial, implicating genetic susceptibility, environmental factors, infectious agents, and autoimmunity. These elements likely sustain an unknown antigen, driving immunopathogenesis through antigen presentation to CD4 + T cells, monocyte activation via inflammatory mediators, and granuloma formation from macrophage fusion into multinucleated giant cells [7].

Presentations vary by organ, with pulmonary symptoms like dry cough, dyspnoea, and fatigue being most common [8]. Renal sarcoidosis is rarer, with incidence estimates of 6% to 48% in sarcoidosis cases, potentially explained by inconsistent screening and diverse manifestations [9, 10]. Renal involvement can lead to significant morbidity, including acute kidney injury (AKI), chronic kidney disease (CKD), and end-stage kidney disease (ESKD) [11]. Key mechanisms of renal injury include aberrant calcium homeostasis [12], where granuloma-produced 1-alpha hydroxylase elevates 1,25-dihydroxyvitamin D which enhances intestinal calcium absorption, bone resorption, and renal reabsorption. This results in hypercalcemia with suppressed parathyroid hormone (PTH) and hypercalciuria leading to a reduced glomerular filtration rate (GFR) through afferent vasoconstriction, dehydration, and an increased risk of nephrolithiasis, nephrocalcinosis, tubulointerstitial inflammation, and acute tubular necrosis (ATN). Acute effects are often considered reversible, but chronic exposure leads to irreversible fibrosis and CKD.

Renal sarcoidosis may mimic other causes of tubulointerstitial nephritis or glomerulonephritis, making biopsy essential for diagnosis. Histologically, granulomatous interstitial nephritis (GIN) is predominant, presenting as AKI or CKD. Other lesions can include various forms of glomerulonephritis [13, 14].

Despite recognition of extrapulmonary sarcoidosis, renal involvement is underrepresented in the literature, with limited prospective data on optimal management. In this study, we aimed to: [1] characterize the clinical presentation, histological patterns, and treatment responses in biopsy proven renal sarcoidosis [2], identify predictors of adverse renal outcomes, and [3] synthesize findings with existing literature to inform clinical management and highlight areas requiring further investigation.

## Methods

This study consists of two components: [1] a retrospective observational cohort study of biopsy proven renal sarcoidosis from Manchester, UK, and [2] a narrative literature review synthesizing published data on renal sarcoidosis to contextualize our findings.

### Retrospective case series

#### Study design and setting

We performed a retrospective observational study at Manchester University NHS Foundation Trust (MFT). The study included consecutive adults evaluated by nephrology service with biopsy-proven kidney disease attributed to sarcoidosis between 2008 and 2022.

#### Inclusion criteria

Age ≥ 18 years; kidney biopsy demonstrating GIN, tubulointerstitial nephritis (TIN) compatible with sarcoidosis, nephrocalcinosis with supportive systemic sarcoid features, or glomerular disease with concurrent systemic sarcoidosis and no alternative aetiology. Systemic sarcoidosis was defined according to international consensus criteria requiring: [1] histological evidence of non-caseating epithelioid cell granulomas from at least one organ [2], compatible clinical and radiological features, and [3] exclusion of other causes of granulomatous disease, including mycobacterial and fungal infections, malignancy, and drug reactions. Patients in whom another primary renal diagnosis explained the presentation were excluded.

#### Data

We extracted demographics (age, sex), sarcoidosis history (age at sarcoid diagnosis, organ systems involved), kidney parameters at presentation (serum creatinine, estimated glomerular filtration rate [eGFR], urine protein to creatinine ratio [uPCR], dialysis requirement), histopathology (GIN/TIN, glomerular lesions, interstitial fibrosis where reported), treatment (initial steroid use and dose category; adjunctive immunosuppression), and outcomes. Laboratory values were recorded at presentation to nephrology and at 1, 3 and 12 months after treatment initiation, and at last follow-up (FU). Data completeness was > 95% for core variables (eGFR, uPCR, histology, treatment).

#### Exposure definitions

‘High-dose’ prednisolone referred to ≥ 30 mg/day at initiation. Steroid-sparing therapy included azathioprine (AZA), mycophenolate mofetil (MMF), methotrexate (MTX), calcineurin inhibitors (CNI) or biologics.

#### Outcomes

The primary outcome was a composite outcome: development of CKD stage 4 or 5 (eGFR < 30 mL/min/1.73 m² sustained ≥ 3 months) or progression to ESKD requiring kidney replacement therapy. Secondary outcomes were eGFR trajectories at 1, 3, 12 months and last FU; relapse of renal sarcoidosis (clinician-adjudicated recurrent kidney dysfunction attributable to sarcoidosis requiring escalation of therapy); and all-cause mortality. For patients progressing to ESKD requiring renal replacement therapy, eGFR values after dialysis initiation were not included in subsequent summary analyses.

### Statistical analysis

Given the small sample size (*n* = 32) and potential for non-normal distributions, all continuous variables are reported as median [interquartile range]. Comparisons between outcome groups were conducted using T test for continuous variables and Fisher’s exact test for categorical variables. A p-value < 0.05 was considered statistically significant. Cox proportional hazards models were used to identify predictors of the primary outcome. Kaplan–Meier curves were generated and plotted for ESKD-free and relapse-free survival with risk tables and 95% confidence intervals. Data analysis was performed using R version 4.4.1 (2024-06-14).

### Narrative review

We conducted a structured literature search of PubMed and Scopus with keywords “sarcoidosis” or “sarcoid” combined with “renal” or “kidney.” Titles and abstracts were screened for relevance and for biopsy proven kidney sarcoidosis. We included prospective and retrospective clinical studies reporting renal manifestations, histology, treatments, or outcomes, and excluded single case reports and narrative reviews without original data. Data extracted included study characteristics, demographics, baseline renal function, histology, treatments (steroids, immunosuppression), and reported outcomes. Studies were summarised in tabular form to contextualise findings from our cohort. This review was conducted to provide representative contextual synthesis and did not follow formal systematic review of PRISMA methodology.

## Results

### Case series, manchester

#### Characteristics

Thirty-two patients met inclusion criteria (Table 1); median age was 61.5 [50.8,70.2] years and 19 (59.4%) were male. Kidney disease was part of the initial presentation of sarcoidosis in 14/32 (43.8%). Among those with pre-existing sarcoidosis, the median interval from systemic diagnosis to kidney biopsy was 2.5 [1.0, 8.8] years. At the time of renal evaluation, 24 (75.0%) had extra-renal involvement. Diabetes mellitus was recorded in 5 (15.6%) patients at presentation. Median follow up for the whole cohort was 6.0 [4.0, 10.0] years.

**Table 1:** Baseline characteristics of patients stratified by Primary outcome

*n*		Overall	No	Yes	*p*
		32	23	9	
Age (median [IQR])		61.5 [50.8, 70.2]	63.0 [53.5, 69.5]	55.0 [30.0, 70.0]	0.201
Male, n (%)		19 (59.4)	15 (65.2)	4 (44.4)	0.427
Age at Sarcoidosis diagnosis (median [IQR])		51.0 [35.5, 59.5]	52.0 [47.5, 60.0]	32.0 [20.0, 54.0]	0.056
Age at time of kidney biopsy (median [IQR])		52.5 [36.0, 62.0]	53.0 [49.0, 61.5]	47.0 [19.0, 62.0]	0.294
Known Sarcoidosis prior to kidney biopsy (%)		14 (43.8)	9 (39.1)	5 (55.6)	0.453
Time from Sarcoid diagnosis to kidney biopsy, years (median [IQR])		2.5 [1, 8.8]	2 [1, 5]	5 [3, 13]	0.172
Other organ Involvement, n (%)		24 (75)	19 (82.6)	5 (55.6)	0.176
Lung, n (%)		17 (53.1)	14 (60.9)	3 (33.3)	0.251
Eye, n (%)		11 (34.4)	9 (39.1)	2 (22.2)	0.365
Skin, n (%)		3 (9.4)	2 (8.7)	1 (11.1)	0.176
Joints, n (%)		2 (6.2)	1 (4.3)	1 (11.1)	0.140
Liver, n (%)		1 (3.1)	1 (4.3)	0	
Lymph nodes, n (%)		3 (9.4)	3 (13)	0	
Parotid, n (%)		1 (3.1)	0	1 (11.1)	
Known DM, n (%)		5 (15.6)	2 (8.7)	3 (33.3)	0.121
eGFR at Sarcoidosis diagnosis (median [IQR])		53.5 [40, 94.8]	53 [41, 99]	68 [28, 77]	0.489
eGFR at presentation to nephrologist (median [IQR])		29.5 [19.5, 56.8]	43 [23, 68.5]	20 [11, 28]	0.049
uPCR at presentation to nephrologist (median [IQR]) mg/mmol		46 [25.3, 73]	37.5 [16, 63.5]	69.5 [46.5, 127.3]	0.096
Dialysis required at presentation to nephrologist, n (%)		2 (6.2)	1 (4.3)	1 (11.1)	1.000
Biopsy features, n (%)	GIN	19 (59.4)	14 (60.9)	5 (55.6)	0.564
	TIN	6 (18.8)	5 (21.7)	1 (11.1)	
	TIN + FSGS	1 (3.1)	1 (4.3)	0 (0.0)	
	IgAN	2 (6.2)	1 (4.3)	1 (11.1)	
	MN	1 (3.1)	1 (4.3)	0	
	CGN	1 (3.1)	0	1 (11.1)	
	ATN	1 (3.1)	0 (0.0)	1 (11.1)	
	Non-specific	1 (3.1)	1 (4.3)	0 (0.0)	
Steroids as initial treatment, n (%)		25 (78.1)	17 (73.9)	8 (88.9)	0.640
Initial treatment, n (%)	Pred	22 (68.8)	16 (69.6)	6 (66.7)	0.192
	CsA	1 (3.1)	1 (4.3)	0	
	Infliximab	1 (3.1)	1 (4.3)	0	
	Methotrexate	1 (3.1)	0	1 (11.1)	
	MP	2 (6.2)	1 (4.3)	1 (11.1)	
	No	4 (12.5)	4 (17.4)	0	
	Pred + CsA	1 (3.1)	0 (0.0)	1 (11.1)	
Prednisolone dosing, n (%)	High	18 (56.2)	13 (56.5)	5 (55.6)	0.305
	Low	3 (9.4)	1 (4.3)	2 (22.2)	
	NA	11 (34.4)	9 (39.1)	2 (22.2)	
Relapse, n (%)		10 (31.2)	5 (21.7)	5 (55.6)	0.096
Months to Relapse (median [IQR])		25 [14.5, 30.75]	16 [14, 26]	32 [24, 43]	0.209
Steroid Sparing Post Relapse, n (%)	AZA	4 (12.5)	2 (8.7)	2 (22.2)	0.128
	CsA	1 (3.1)	1 (4.3)	0	
	Methotrexate	1 (3.1)	1 (4.3)	0	
	No	4 (12.5)	1 (4.3)	3 (33.3)	
	NA	22 (68.8)	18 (78.3)	4 (44.4)	
ESKD, n (%)		4 (12.5)	0	4 (44.4)	
Months to ESKD (median [IQR])		6.5 [4.8, 18]	NA	6.5 [4.8, 18]	
Follow up in Years (median [IQR])		6 [4, 10]	7 [5, 10]	4 [2, 6]	0.135
Death, n (%)		3 (9.4)	2 (8.7)	1 (11.1)	1.000

DM, diabetes mellitus; GIN, granulomatous interstitial nephritis; TIN, tubulointerstitial nephritis; FSGS, focal segmental glomerulosclerosis; IgAN, immunoglobulin A nephropathy; MN, membranous nephropathy; CGN, chronic glomerulonephritis; ATN, acute tubular necrosis; NA, not available; Pred, prednisolone; CsA, cyclosporine A; MP, methylprednisolone; AZA, azathioprine; ESKD, end stage renal disease. ESKD occurred only in the primary outcome group by definition; time to ESKD is reported only for affected patients

#### Presentation and histopathology

At presentation to nephrology service, median eGFR was 29.5 [19.5, 56.8] mL/min/1.73 m² and uPCR was 46 [25.3, 73.0] mg/mmol. Baseline median eGFR at diagnosis of sarcoidosis in those without prior kidney involvement was 53.5 mL/min/1.73 m² and 30 mL/min/1.73 m² when diagnosed with renal sarcoidosis. Two patients (6.2%) required dialysis at presentation. Kidney biopsy showed GIN in 19 patients (59.4%). Other patterns included TIN without granulomas (6, 18.8%), IgA nephropathy (IgAN) (2, 6.2%), TIN + focal segmental glomerulosclerosis (FSGS) (1, 3.1%), membranous nephropathy (MN) (1, 3.1%), crescentic GN (1, 3.1%), and ATN (1, 3.1%). Biopsy showed only non-specific changes in 1 patient.

#### Treatment

Initial corticosteroid therapy was administered in 25/32 patients (78.1%): oral prednisolone in 22, pulse intravenous methylprednisolone in 2, and combination prednisolone plus CsA in 1 patient. High-dose prednisolone was documented in 18 patients and low-dose in 3 (dose unavailable in 4). Three patients received steroid-sparing immunosuppression as initial therapy (CsA *n* = 1, MTX *n* = 1, infliximab *n* = 1), 2 were on disease modifying treatment for extra-renal sarcoidosis, and 2 did not receive any treatment (1 had IgA nephropathy without significant proteinuria and another patient had mild non-specific changes). When steroid-sparing agents were introduced during follow-up, these included MMF (*n* = 2), AZA(*n* = 1), MTX (*n* = 1) and infliximab (*n* = 1).

#### Outcomes, relapse and mortality

**Fig. 1:**
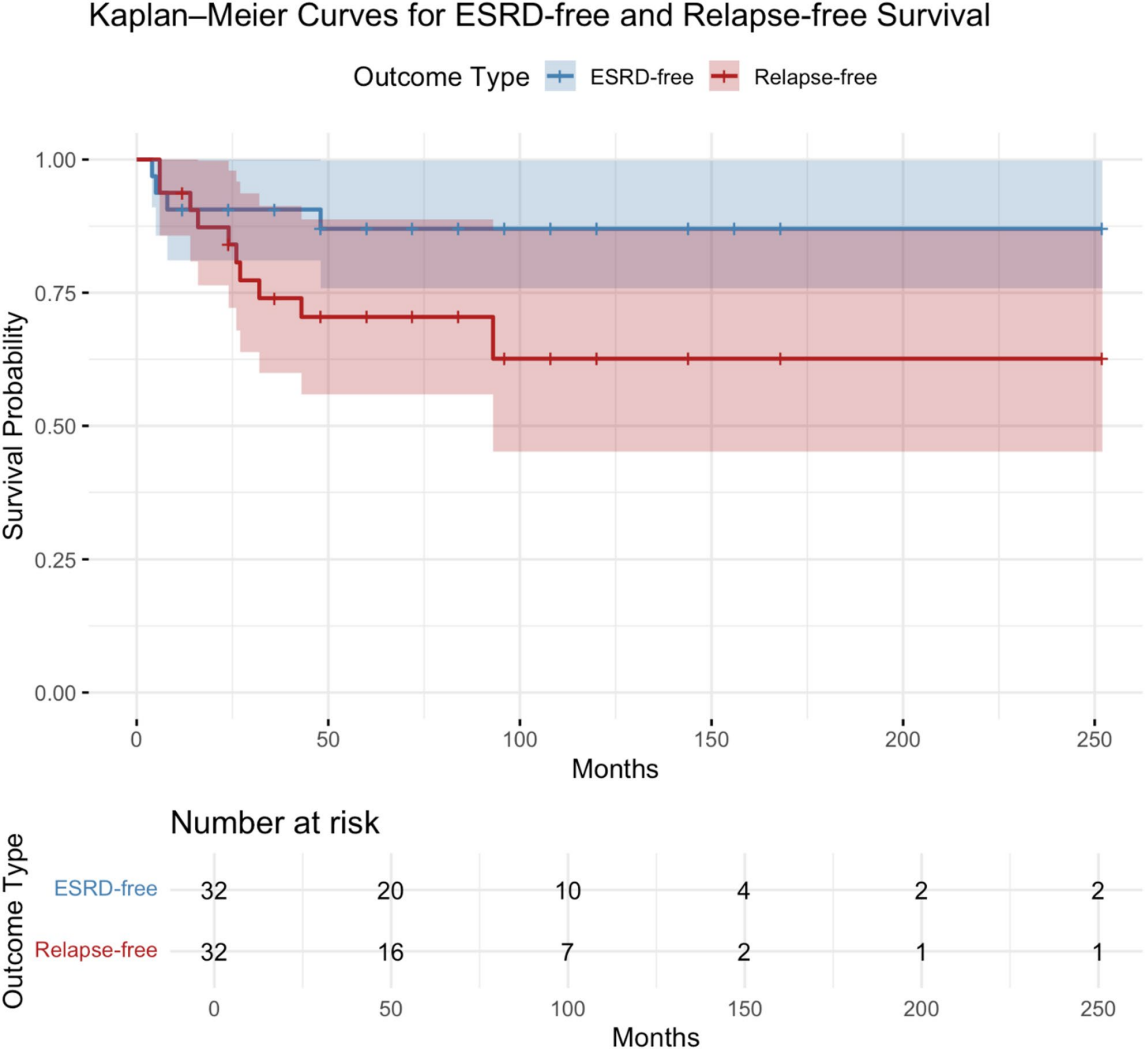
Kaplan-Meier curves of ESKD-free and relapse-free survival

Recovery of kidney dysfunction was partial in majority of patients (Fig. 1; Table 2). 9 patients (28.1%) experienced primary outcome. ESKD occurred in 4 patients (12.5%) at a median of 6.5 [4.8,18.0] months. 10 patients (31.2%) relapsed at 25 [14.5, 30.8] months, all received treatment with steroids. Post relapse steroid sparing therapy included AZA (*n* = 4), CsA (*n* = 1), and MTX (*n* = 1); 4 patients received no additional steroid sparing agent. 3 patients (9.4%) died during follow-up.

**Table 2:** eGFR trends according to outcome (CKD stage 4 or 5 or progression to ESKD) status

eGFR at 1 month (median [IQR])	Overall	No Primary Outcome	Primary Outcome	*p*
	39 [27.25, 55.75]	47 [34.5, 63.75]	22.5 [14, 36.25]	0.008
eGFR at 3 months (median [IQR])	41.5 [30, 49.75]	44 [38, 51]	28 [17, 35]	0.016
eGFR at 12 months (median [IQR])	43.5 [33.75, 61.25]	45 [42, 65]	29 [23, 37.5]	0.007
eGFR at Last FU (median [IQR])	48 [26.5, 58]	53.5 [47.25, 62.25]	16 [8, 21]	< 0.001

Primary outcomes: a composite adverse outcome: development of CKD stage 4 or 5 (eGFR <30 mL/min/1.73 m² sustained ≥3 months) or progression to ESKD requiring kidney replacement therapy. FU, follow up. eGFR values after initiation of renal replacement therapy were excluded from summary statistics

#### Renal trajectories

**Fig. 2:**
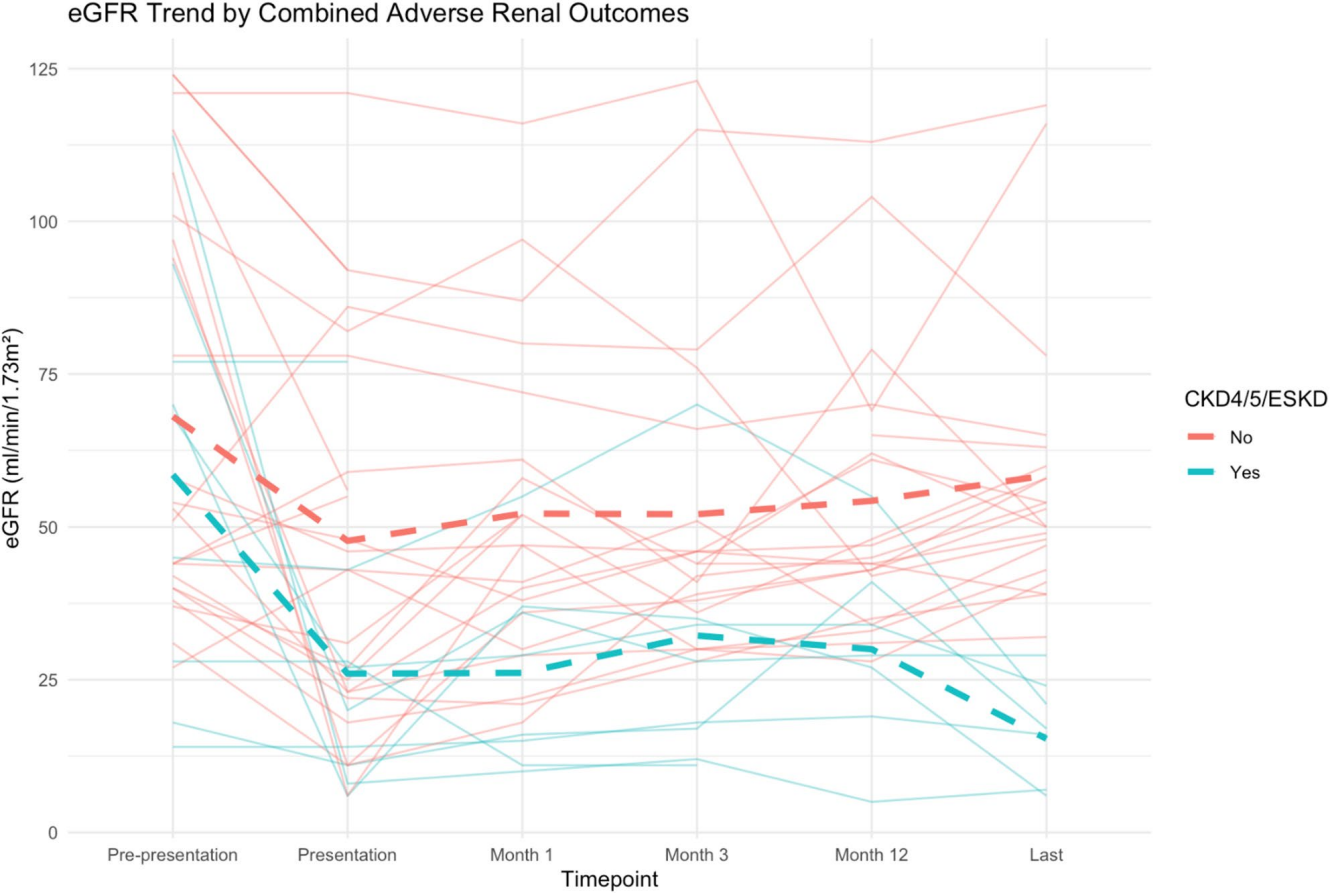
Longitudinal trends in eGFR by primary outcome status. Individual patient trajectories are show in thin lines, group means are shown in thick dotted lines

Kidney function improved rapidly after treatment and then stabilised. eGFR increased from 29.5 to 39.0 [27.3, 55.8] mL/min/1.73 m² at 1 month, 41.5 [30.0, 49.8] at 3 months, reaching 43.5 [33.8, 61.3] by 12 months. At last follow-up, eGFR was 48 [26.5, 58] mL/min/1.73 m², consistent with partial recovery for the cohort overall (Fig. 2).

Among the 25 patients who received initial steroid therapy, histological patterns included GIN (*n* = 16), TIN without granulomas (*n* = 6), glomerular lesions (TIN+FSGS and CGN, *n* = 2) and ATN (*n* = 1). Patients with GIN showed significant improvement in eGFR from presentation to 1 month (23 [11, 32] to 37 [20,47] mL/min/1.73m^2^, *p* = 0.008), with sustained improvement at months 12 and last follow up (39 [29,47] and 45 [20,55] mL/min/1.73m^2^ respectively). 3 of 16 (18.8%) progressed to ESKD. Patient with TIN without granuloma had better baseline eGFR (46 [27,81] mL/min/1.73m^2^) and showed variable response to treatment, with no progression to ESKD.

#### Predictors of primary outcome

**Fig. 3:**
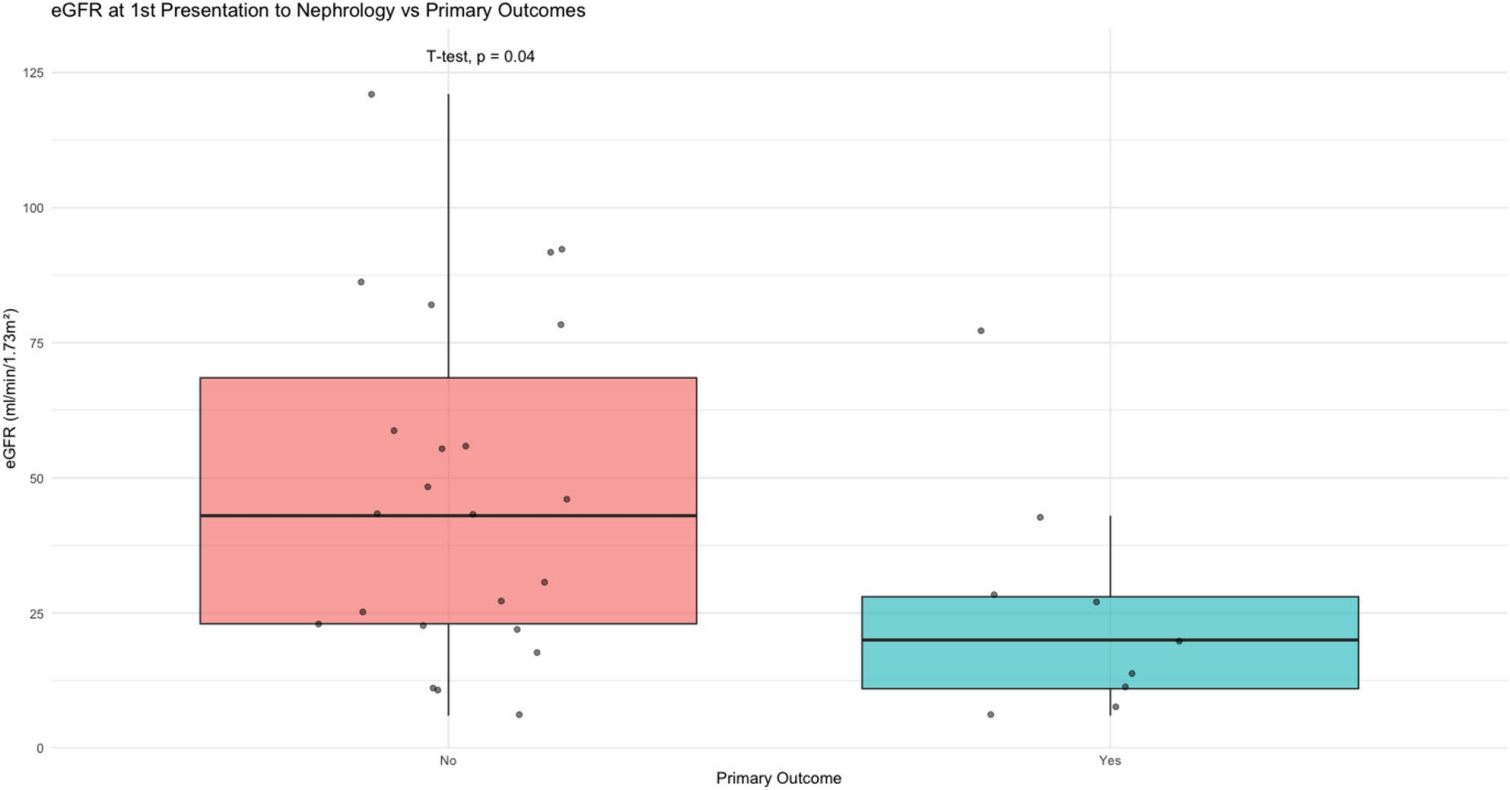
Box plot comparing eGFR at first presentation to Nephrology between patients with and without subsequent primary outcome status (T-test, *p* = 0.04)

Patients who developed primary outcome during follow up presented with more advanced renal impairment: eGFR 20 vs. 43 mL/min/1.73 m² (*p* = 0.049, Fig. 3) and numerically higher uPCR (69.5 vs. 37.5 mg/mmol; *p* = 0.101). There were no significant differences in the distribution of histopathological categories or in the proportion receiving high-dose steroids. Subsequent eGFR remained consistently lower in the primary outcome group during the 12 months of initial follow up period, with last eGFR being 16.0 vs. 53.5 mL/min/1.73 m² (*p* = 0.001). In univariate Cox analysis, younger age at diagnosis was associated with primary outcome (HR 0.95, 95% CI 0.91–1.00; *p* = 0.039), with concordance 0.671 and likelihood ratio *p* = 0.034.In sensitivity analysis excluding patients with baseline CKD stage 4/5 (*n* = 4), the association between younger age at diagnosis and primary outcome remained unchanged (HR 0.94, 95% CI 0.89–0.996; *p* = 0.036). Relapse status carried wide uncertainty due to few events.

## Discussion

In the single centre cohort of biopsy proven renal sarcoidosis we observed that in most patients presenting with impaired kidney function, granulomatous interstitial nephritis (GIN) was the predominant lesion, and early recovery occurred following corticosteroid therapy, particularly in patients with GIN. Nevertheless, 28% developed the primary outcome (CKD stage 4/5 or ESKD), baseline renal impairment and younger age at diagnosis were associated with worse prognosis, these findings require cautious interpretation in view of limited sample size.

Our findings align with prior studies demonstrating that renal involvement in sarcoidosis is characterised by diverse clinical and histological manifestations (Table 3). The most common histopathological lesion is GIN. In addition to GIN, sarcoidosis is associated with a spectrum of other renal pathologies, including nephrocalcinosis, TIN without granulomas, and a variety of glomerular diseases such as MN, which was seen to be the most frequent glomerular lesion [13]. Other glomerular lesions included IgAN, FSGS, minimal change disease (MCD), and lupus nephritis [13, 15, 16]. Baseline renal function is often impaired, with eGFR ranging from 14 ml/min to 74 ml/min [15, 17]. eGFR at presentation was comparable between de novo and previously diagnosed sarcoidosis cases, suggesting that renal involvement may go unrecognised until a similar degree of kidney dysfunction develops. Finally, one study suggests acute kidney injury is notably common among those with GIN [15].

**Table 3:** Summary of key studies on renal sarcoidosis (1978–2025)

Author, Country, Year	Study type	Study group	Demographic	Kidney histology	Treatment	Outcome	Comments
MacSearraigh et al. Ireland, 1978 [24]	Retrospective single centre	*N* = 9	Age- mean 22 years 7 M/2F eCrCl – median 45 Proteinuria – median 2.6 g/day	• Kidney histology in 8 out of 9 patients • GIN *n* = 5 • TIN without granulomas *n* = 1 • MPGN *n* = 1 • Nephrocalcinosis *n* = 4	• 8 out of 9 received corticosteroids	• ESKD *n* = 1 • Death *n* = 1	• No information on treatment, duration, follow up length or effects on kidney function
Robson et al. UK, 2003 [17]	Retrospective single centre	*N* = 7 Biopsy proven GIN in the absence of extrarenal sarcoidosis	Age- mean 69 years 5 M/2F eCrCl – median 14mL/min Proteinuria – median 0.42 g/day	• GIN 100%	• Initial prednisolone median dose of 40 mg/day • 1 received IV MP	• 5/7 improved renal function • 2/7 progressed to ESKD despite treatment	• No statistical analysis of data
Joss et al. UK, 2007 [25]	Retrospectove single centre (1990–2004)	*N* = 5 (5 biopsy proven GIN out of 18 with GIN)	Age – median 64 years 3 M/2F eCrCl – median 22 Proteinuria - NA	• 100% GIN	• Steroids *n* = 5 • Steroid sparing immunosuppression *n* = 3	• CrCl improved from 4–60 mL/min pretreatment to 29–94 mL/min at last follow up	• Mean follow up was 45 months • Prednisolone was used for 36 months on average
Javaud et al. France, 2007 [20]	Retrospective multicentre (7 centres, 1991–2004)	*N* = 20 (out of 40 patients with renal granulomatosis)	Age – median 53.5 years M12/F8 eCrCl – median 26 Proteinuria – median 0.45 g/day	• GIN 100% • Concomitant vasculitis	• 3 received IV MP for 3 days • Others, induction therapy of 1 mg/kg for median of 30 days • Long term steroids for median of 21.5 months	• eCrCl 26mL/min improved to 46.8mL/min • 1 patient with ESKD and 1 death	• Median follow up 25.5 • 50% of patients with sarcoidosis had acute renal insufficiency, the other 50% were chronic
Mahevas et al. France, 2009 [22]	Retrospective multicentre (3 centres, 1988–2007)	*N* = 47	Age – median 47 years M30/F17 eGFR – median 20.5 Proteinuria – median 0.7 g/day	• GIN *n* = 37 • TIN without granulomas *n* = 10 • Concomitant IgA deposits *n* = 2	• Prednisolone 100% • 1 mg/kg/day *n* = 39 • 0.7 mg/kg/day *n* = 6 • 0.5 mg/kg/day *n* = 2 • 10 received IV MP • Median duration of prednisolone 18 months • In 6/46 (13%), steroids were permanently stopped, with 5 considered disease free • 24/41 cases where steroid therapy continued at the end of follow up had no relapse	• Mean increase in eGFR from 20 ± 19.1 mL/min to 44 ± 24.7 mL/min after 1-month steroid therapy • Similar levels of complete, partial and unfavourable response at 1 month vs. 1 year • No significant difference in eGFR at 1 year follow up vs. 1 month	• An inverse relation between response to steroid therapy and initial degree of interstitial fibrosis on biopsy • Complete response to therapy at 1 year and last follow-up strongly correlated with compete response at 1 month.
Aouizerate et al. France, 2010 [23]	Retrospective multicentre (8 centres, 1992–2007)	*N* = 18 (Patients with sarcoidosis who underwent renal transplantation)	Age – mean 43.5 ± 11 years M12/F6 Mean eGFR – 60 (± 25) mL/min/1.73 m²;	• GIN related to sarcoidosis *n* = 10	• Renal transplantation in 18 patients • Induction immunosuppression in 11 patients (4 rabbit anti-thymocyte globulin, 7 IL-2 antagonist) • Maintenance – CNI 18, sirolimus 2, corticosteroids 16, mycophenolate / azathioprine 16	• Mean eGFR at last follow up 60 ± 25 mL/min • Mean patient survival and death censored graft survival were 94.4% • Recurrence of sarcoidosis in 5, with 4 of the 5 having renal sarcoidosis as the primary renal disease	• Mean follow up 4 years • Recurrence occurred within the first 18 months for 4 out of 5 patients • Primary renal sarcoid strongly associated with recurrence • Suggests longer time between last sarcoid episode and transplant reduces risk of recurrence
Stehle et al. France, 2013 [13]	Retrospective multicentre (7 centres, 1977–2012)	*N* = 26	Age – mean 37 years M18/F8 eGFR – mean 70.7mL/ min/1.73 m²; Proteinuria – mean 5.7 g/day	• Membranous nephropathy, *n* = 11 (42%) • IgAN *n* = 6 (23%), FSGS *n* = 4, mcd *n* = 3, lupus nephritis *n* = 2 • Concurrent GIN *n* = 6 23% pre-diagnosis, 42% post sarcoidosis diagnosis, 35% simultaneously	• Steroid cytotoxic treatment, *n* = 18 (69%) • No association of remission to steroids and glomerular disease	• Follow up 8.4 years • ESKD *n* = 6 (23%) • In other 20 patients, mean eGFR 67.8 mL/min • Death *n* = 3 (12%)	• Suggests heterogeneity in glomerular disease in sarcoidosis • Rare for complete remission using steroid therapy exclusively
Bagnasco et al. USA, 2014 [26]	Retrospective single centre (2000–2011)	*N* = 10 (19 confirmed renal sarcoidosis on biopsy out of 51 patients with a history of sarcoidosis- only 10 of these patients had follow-up data)	Age – median 49 years M5/F5 Serum creatinine: 3.96 ± 2.35 mg/dl Proteinuria − 0.94 ± 0.65 g/24 hours	• GIN *n* = 19 (37%) • TIN without granuloma *n* = 8 • FSGS *n* = 6, immune complex GN *n* = 3, MN *n* = 1 • Others: 19 (including 6 with advanced changes, and 7 with diabetic nephropathy)	• Prednisolone: mean 50 mg/day (only available in 10 patients) • Total duration of treatment – mean 1.5 years	• 1 out of 10 developed ESKD	• 3–5% of patients with renal sarcoidosis progressed to ESKD • Estimated less than 1 in 1000 patients receiving transplanted kidney is due to sarcoidosis as a primary cause of ESKD
Loffler et al. Germany, 2015 [14]	Retrospective Single Centre (2000–2013)	*N* = 27	Age- mean 55 years M: F = 1.7:1 Baseline eGFR 38 ± 21 mL/min; proteinuria 981 ± 304 mg/24 h	• GIN (30%) • TIN (44%) • IgAN (26%)	• Oral Prednisolone	• eGFR 38→57 mL/min; • Proteinuria decreased 981→176 mg/24 h; • 62.5% responded;	• GIN associated with more advanced renal insufficiency • Eearly treatment response is key prognostic factor
Kamata et al. Japan, 2018 [18]	Retrospective multicentre (5 centres, 2000–2015)	*N* = 16	Age – mean 59.4 years M7/F9 eGFR - mean 28.2 ± 16.1 mL/ min/1.73 m²; Proteinuria > 300 mg/day, *n* = 10 (63%)	• Granulomatous TIN in 13 (81%) • TIN without granuloma in 3 (13%) • Renal calcinosis in 2 (12%) • FSGS 1 (6%) and HTN 1 (6%)	• Dose of steroid • Mean dose at initiation 0.5 mg/kg/day • Mean period of steroids 3.5 years • Steroid sparing therapy azathioprine (*N* = 1)	• Change in eGFR • 28.1+/- 15mL/min to 43.5 +/- 20.4 mL/min • Reduction in stage 4/5 CKD but residual renal dysfunction in 80%	• Favourable response to steroid therapy in 60% • Favourable response with initial loading dose of 0.6 mg/kg/day • Unfavourable response in those with loading dose of 0.4 mg/kg/day
Naderi et al. Germany, 2019 [21]	Retrospective single centre	*N* = 7	Age – median 67 years M6/F1 eGFR – mean 19 ± 7 mL/min Proteinuria – mean 1.9 g/day	• 5 patients with GIN, 1 with interstitial nephritis without granuloma, 1 with nephrocalcinosis • 1 had concomitant MN and focal proliferative GN, 1 had concomitant IgAN	• Patients received oral prednisolone, either 1 mg/kg (*n* = 4) or 0.5 mg/kg (*n* = 3) daily	• Mean eGFR improved from 19 ± 7 to 49 ± 16 mL/min • No difference in renal function improvement in the two groups	• Mean follow up 59 months • All patients on higher dose oral prednisolone developed diabetes mellitus
Zammouri et al. Tunisia, 2019 [27]	Retrospective Single centre (1978–2015)	*N* = 24	Age mean- 46.3 ± 15.1 years 22 F/2 M Median eGFR 14.3 mL/min/1.73 m²; median Cr 313 µmol/L; proteinuria 0.68 ± 0.26 g/24 h	• GIN (43%) • TIN (57%)	• Oral Prednisolone • 2 received cyclophosphamide	• Median eGFR 14→32 mL/min; • 4/24 progressed to ESKD;	• Predictors of ESKD: multiorgan involvement (> 3 organs), advanced fibrosis (F3), extensive interstitial granulomas • Response at 6 months predicts long-term outcome
Frelih M et al. Slovenia, 2021 [28]	Retrospective national registry (2010–2020)	*N* = 13 (7 sarcoidosis, 6 TINU)	Age- mean 60 years 9 F/4 M indications: AKI (*n* = 8), acute on CKD (*n* = 4), proteinuria (*n* = 1)	• GIN	• Corticosteroids	• 11/13 (85%) improved kidney function at 6 months; • Proteinuria decreased in 9; • 1 remained on dialysis	• Mixed cohort includes TINU syndrome (*n* = 6) separate from sarcoidosis (*n* = 7); • Favourable prognosis with timely treatment
Rastelli et al. Italy 2021 [15]	Retrospective multicentre (36 centres, 2013–2019)	*N* = 39	Age – median 60.5 years M28/F11 eGFR – median 74 mL/ min/1.73 m²; Proteinuria – 0.84 g/day	• 31 GIN (sGIN) • 3 TIN without granulomas • 1 tubular microcalcification • 1 chronic lesion • 1 GIN _ FSGS • 1 MCD • 1 Fibrillary GN	• 1 mg/kg/day (*n* = 20), 0.5–0.8 mg/kg/day (*n* = 5), 0.5 mg/kg/day (*n* = 9), others variable doses • 9 also received IV MP (doses unavailable)	• Complete recovery in 5 (100%) non-s-GIN-AKI, 45% in GIN-AKI • 65% (13/20) of patients with normal basal renal function developed CKD by the end of follow up	• 86% of patients had sGIN-AKI with AKI being more severe in this group of patients • In acute phase of sarcoid GIN as a cause of AKI, steroid therapy will prevent deterioration to CKD in only 1/3 of cases
Gorsane et al. Tunisia, 2022 [16]	Retrospective single centre (1976–2017)	*N* = 34	Age – mean 47.1years M6/F28 eGFR – median 16.5 mL/ min/1.73 m²; Proteinuria – 0.65 g/day	• Kidney biopsy *n* = 17 • TIN *n* = 14 • Glomerular disease *n* = 3 • MN *n* = 2 • IgAN *n* = 1	• Oral corticosteroids in 33 patients • 0.5 mg/kg/day *n* = 17 • 1 mg/kg/day *n* = 16 • Average duration of initial corticosteroid therapy was 4.73 ± 1.45 weeks • Maintenance steroid therapy for 23.02 ± 14.45 months • Steroid sparing immunosuppression • Azathioprine *n* = 1 • Cyclophosphamide *n* = 2	• With treatment, the median GFR increased from 16.5 at baseline to 23.1 at 1 month and 34.1 at 6 months to 41.1 at 1 year follow up • Final progression to end stage chronic renal disease was noted in 8 patients (23.5%) • Overall renal survival was 82.3% at 1 year and 76.4% at 5 years	• Mean follow up of 79.2 ± 25.7 months
Bergner et al. Germany, 2023 [29]	Retrospective multicentre registry (5 centres, 2001–2021)	*N* = 327 total (109 suspected renal sarcoidosis (90 biopsy (27.5%) proven renal sarcoidosis), 197 non-renal sarcoidosis)	Age- median 58 years (renal) vs. 51 (non-renal) 45 F/64 M (renal) 114 F/83 M (non-renal) Median eGFR 41.5 mL/min/1.73 m² (renal) vs. 93 (non-renal); median proteinuria 328 mg/g Cr	• GIN 32.4% • TIN 35.2%	• Steroids 92% (renal) vs. 78% (non-renal); AZA 38% vs. 16%; MMF 5% vs. 1%; MTX 7% vs. 17% (less in renal)	• 64% had eGFR < 60 mL/min/1.73 m²; 57/90 with significant renal impairment; no follow-up data reported	• Largest biopsy-proven cohort
Zhu et al. China, 2023 [30]	Retrospective single centre (2005–2021)	*N* = 18 (15 had kidney biopsy)	Age- mean 48.2 ± 0.4 years 14 M/4F Median eGFR 30.4 [11.6, 60.1] mL/min/1.73 m²; median proteinuria 1.10 [0.69, 1.58] g/24 h	• GIN 66.7% (10/15) • TIN 20% (3/15) • GN with TIN 26.7% (4/15); • Interstitial fibrosis scored 0–3	• Prednisone 0.5-1 mg/kg/day in 17/18; • 5 received pulse MP (500 mg x3d); • 3 with MMF, • 2 with Tripterygium wilfordii glycosides, • 1 with Cyclophosphamide	• Median eGFR 30.4→58.5 at 1 m (*p* < 0.01), 69.5 at end FU; • Proteinuria 1.10→0.68 at 1 m, 0.39 at end FU; • No relapses, • No ESKD; • Median FU 24.1 [8.8, 60.9] months	• 83% had lung involvement, 1 isolated renal sarcoidosis • Early response at 1 month predicts long-term outcome • Eeven fibrosis score 2 responded well to treatment • 8/17 maintained on low-dose steroids at end FU
Mahevas et al. France, 2023 [19]	Multicentre prospective randomised, open label-controlled trial (21 centres, 2012–2016)	*N* = 40	Age – median 60 years M28/F12 eGFR – 22–25 in PRD and MP groups uPCR – 53.7 / 75.7 in PRD and MP groups	• 100% in TIN • Interstitial calcification 25% in PRD and 44% in MP groups • Glomerulosclerosis 19% in PRD and 24% in MP groups • Interstitial fibrosis 20% PRD group, 21% in MP group	• PRD 1 mg/kg/day • MP 12 mg/kg/day for 3 days followed by oral prednisolone 1 mg/kg/day • Prednisolone tapered by 10 mg every 15 days to reach • 0.5 mg/kg/day at 3 months • 0.25 mg/kg/day at 6 months • 5 mg at 12 months	• Primary end point: positive response at 3 months, i.e. doubling of eGFR compared to pre-randomisation or eGFR > 60mL/min • 80% in PRD group vs. 50% in MP group • Median eGFR at 3 months 45 in PRD vs. 46 in MP groups • No difference in severe adverse events	• Follow up 12 months • Pulse methylprednisolone conferred no benefit in patients with TIN • Kidney function response was maximal at 1 month but did not improve afterwards • The initial renal fibrosis was associated with renal outcome
Dirim et al. Turkey, 2024 [31]	Retrospective single centre (2003–2023)	*N* = 23	Age- mean 47 years 14 F/9 M Median baseline eGFR 26.5 [10.8, 41.3] mL/min/1.73 m²; median Cr 2.23 mg/dL; median proteinuria 1 [0.25, 2.56] g/g or g/day	• GIN 56.5% (13/23), • TIN 8.7% (2/23), • Nephrosclerosis with intratubular calcific casts 17.4% (4/23); • Glomerular disease 17.4% (FSGS *n* = 2, MN *n* = 1, AA amyloid *n* = 1); • IFTA graded	• All received prednisone 1 mg/kg/day; • Additional: AZA 20%, MMF 5%, HCQ 10%, RTX 5%, CSA 5%	• Median FU 48 [17, 72] months; • 45% partial response (no complete response); • 45% developed ESKD; no improvement in eGFR overall (*p* = 0.522); • improvement at 1 m in non-glomerular disease (*p* = 0.049); • 25% mortality	• Predictors of better outcome: hypercalcemia at baseline (*p* = 0.03), lower IFTA (*p* = 0.043). • Predictors of poor outcome: glomerular disease (0% response, *p* = 0.043), late diagnosis, irregular follow-up. • Only 34.8% had prior sarcoidosis diagnosis before biopsy • 86.7% had extrarenal involvement • Poor outcomes attributed to late diagnosis and irregular follow-up
Kamata et al. Japan, 2025 [32]	Retrospective national registry (2007–2021)	*N* = 135 (From 55,885 total biopsies (0.24% prevalence)	Age- mean 62 ± 14 years 69 F/66 M Mean eGFR 30.7 ± 15.2 mL/min/1.73 m²; men 32.2 ± 14.1, women 29.2 ± 16.3; mean UPCR 0.50 ± 0.68 g/gCr	• TIN 98.5% (133/135); • GIN not separately quantified. • Concomitant glomerular disease in 9: • Nephrosclerosis (*n* = 4), • IgA nephropathy (*n* = 2), • Diabetic nephropathy (*n* = 2), • Other (*n* = 1); • Renal calcinosis (*n* = 2); • One with Sjögren’s	• No treatment data - registry only captures data at time of biopsy	• No outcome data - cross-sectional study only	• Sarcoidosis 0.24% of all biopsies, 7.2–8.6% of all TIN. • 53.4% had severe renal dysfunction (G4/G5) • Age peak: 60–70 s (later than general sarcoidosis diagnosis) • UPCR negatively correlated with eGFR (*r*=-0.531, *p* < 0.001) and albumin (*r*=-0.352, *p* < 0.001) • Suggests TIN severity relates to renal dysfunction and proteinuria • Albuminuria: A1 25%, A2 49%, A3 25%. Microhaematuria 11%. DM in 13%.

Consistent with other renal cohorts, corticosteroids were the cornerstone of treatment. Oral prednisolone was typically initiated at 0.5 to 1 mg/kg/day [16, 18–22]. In a Japanese renal cohort, higher initial dosing (0.6 mg/kg/day vs. 0.4 mg/kg/day) was associated with improved renal outcomes [18]. However, randomised data in pulmonary sarcoidosis do not support a benefit of higher dose therapy. In the SARCORT trial, 40 mg/day prednisolone was not superior to 20 mg/day for relapse prevention, lung function, or quality of life outcomes [2]. These findings suggest that while higher doses may be considered in severe renal involvement, routine escalation in sarcoidosis overall is not supported by current evidence. Across renal cohorts, steroid therapy was associated with substantial improvement in eGFR, though treatment-related metabolic complications were common at higher doses [21, 22]. A recent French randomised controlled trial compared oral prednisolone alone to a regimen incorporating IV MP pulses and found no additional renal benefit from the IV MP induction. At three months, 80% of patients receiving oral prednisolone alone achieved a positive renal response, compared to 50% in the pulse treatment group [19]. Steroid-sparing immunosuppressive agents such as azathioprine and cyclophosphamide were used selectively, notably in the studies from Tunisia and Japan, although their role remains adjunctive and not well established [16, 18].

Despite early response, long-term renal outcomes were variable. In the Italian study, 65% of patients with normal baseline renal function developed CKD during follow-up [15]. In Tunisia, 23.5% progressed to ESKD [16]. Complete remission was uncommon, particularly in patients with glomerular involvement, as highlighted in the 2013 French cohort, which showed no clear correlation between corticosteroid use and disease remission [13]. Importantly, early treatment response was predictive of long-term outcome; patients demonstrating improvement within the first month of therapy were more likely to sustain this benefit over time [22].

Kidney transplantation remains a viable option in advanced cases. A 2010 French multicentre study reported excellent outcomes among 18 transplant recipients, with a 94.4% patient and death-censored graft survival rate and mean eGFR was 60 (± 25) ml/min/1.73m^2^ at last follow up (median 42 months) [23]. However, recurrence of sarcoidosis occurred in five patients. The risk of recurrence was highest within the first 18 months post-transplant, and a longer remission interval prior to transplantation appeared to reduce this risk.

Several prognostic indicators have emerged across studies evaluating renal sarcoidosis, with histological and clinical features aiding in anticipating therapeutic outcomes. Among these, the presence and severity of interstitial fibrosis at baseline appears to consistently correlate with poorer renal recovery. In the French randomised controlled trial, patients with a higher degree of fibrosis exhibited significantly less improvement in eGFR at follow-up, despite receiving identical corticosteroid regimens, suggesting that fibrotic remodelling may represent a point of irreversibility in disease progression [19]. This observation is also seen in a 2009 multicentre French cohort, where the extent of fibrotic involvement inversely predicted steroid responsiveness, with early improvements in renal function within the first month being strongly predictive of sustained long-term response [22]. Similarly, the 2021 Italian survey found that even among patients presenting with AKI and normal baseline function, progression to CKD occurred in over 60%, with severity of initial presentation and presumed underlying chronicity likely contributing to this decline [15].

Renal sarcoidosis emerges from this review and case series as a variable manifestation with GIN as the core lesion, often compounded by hypercalcemia and glomerular pathologies. Literature and our data highlight impaired baseline function (eGFR 14–74 mL/min/1.73m^2^), AKI frequency, and diverse histology, aligning with multisystem disease complexity. Steroids drive improvements, but the RCT’s no-benefit from IV MP suggests oral regimens suffice initially. Higher doses correlate with better responses but increase risks like diabetes, as in Germany (100% incidence at 1 mg/kg/day). Adjuncts are underused, yet our relapse rate (31%) suggests need for biologics in refractory cases.

Outcomes reveal partial recovery, with CKD/ESKD in 23–65%, mirroring our 28% adverse rate. Fibrosis as a poor predictor is consistent; our younger age association (HR 0.94) may indicate aggressive phenotypes, warranting age-stratified approaches. Transplantation success (94% survival) contrasts with recurrence risks, emphasising remission timing.

This synthesis reinforces early biopsy and close monitoring to prevent irreversible fibrosis but highlights absence of formalised diagnostic and treatment guidelines. Although no consensus guidelines currently exist, structured approaches to diagnosis and management have been proposed. Hilderson et al. outlined a stepwise framework emphasising histological confirmation, assessment of calcium metabolism, exclusion of alternative causes of granulomatous nephritis, and a staged therapeutic strategy beginning with corticosteroids and escalating to steroid sparing or biologic agents in refractory disease [12]. However, these recommendations derive largely from retrospective series and expert opinion. Prospective registries with harmonised diagnostic criteria, longitudinal biomarker assessment, and embedded randomised trials are needed to move from consensus-based to evidence-based care. Biorepositories for genomics/proteomics could identify susceptibility genes and antigens, informing targeted therapies. Multi-centre RCTs within registries could test additional regimens (adjunctive treatments to reduce steroid exposure including anti-TNF or newer agents), improving personalisation and reducing CKD burden.

### Limitations

We acknowledge that this single centre retrospective study has several limitations. Small sample size precluded multivariable modelling beyond exploratory Cox regression and limited subgroup analyses. Biopsy confirmed cases likely represent more severe phenotypes, potentially underestimating subclinical and milder renal involvement. Haematuria and serum calcium levels were not systematically extracted for analysis. Key methodological limitations include lack of systematic interstitial fibrosis and tubular atrophy (IFTA) quantification in pathology reports, individualised rather than protocol driven treatment decisions, and relapse definitions based on clinical criteria rather than histological confirmation. Our primary outcome definition (CKD 4/5 or ESKD) inherently incorporates renal function and therefore carries the risk of circularity. However, sensitivity analysis excluding patients with CKD stage 4/5 at baseline yielded similar results, mitigating this concern. Lower pulmonary involvement compared to general sarcoidosis cohorts might reflect selection bias inherent to our kidney biopsy cohort. Finally heterogeneous inclusion criteria and lack of standardised definitions for disease activity across the literature complicate comparison with other studies and limit determination of optimal treatment strategies.

Although retrospective and single-centre, this cohort represents one of the larger biopsy-confirmed series with extended follow-up. This case series and review of the literature collectively reinforce the necessity of early diagnosis and biopsy-guided treatment, particularly before irreversible fibrotic changes set in. They also highlight a major gap in clinical guidance; while corticosteroids remain the mainstay of therapy, stratifying patients based on risk of progression and likelihood of response remains an area that requires further prospective validation.

## Conclusions

Renal sarcoidosis is an under recognised, potentially reversible cause of kidney dysfunction in systemic sarcoidosis, with GIN predominance and steroids key for recovery. Incomplete recovery and relapse are common, while the relationship with younger age requires further validation in larger cohorts. Early diagnosis, standardised follow-up, and multicentre and prospective registry data are essential to improve prognosis and inform future therapeutic trials.

## Data Availability

All data generated or analysed during this study are included in this published article.

## Abbreviations

AKI: Acute kidney injury
ATN: Acute tubular necrosis
AZA: Azathioprine
CKD: Chronic kidney disease
CGN: Crescentic Glomerulonephritis
CNI: Calcineurin inhibitors
CsA: Cyclosporin A
eGFR: Estimated glomerular filtration rate
ESKD: End stage kidney disease
FSGS: Focal segmental glomerulosclerosis
FU: Follow-up
GIN: Granulomatous interstitial nephritis
GN: Glomerulonephritis
IFTA: Interstitial Fibrosis and Tubular Atrophy
IgAN: IgA nephropathy
IQR: Interquartile range
MCD: Minimal change disease
MMF: Mycophenolate mofetil
MN: Membranous nephropathy
MP: Methylprednisolone
MTX: Methotrexate
PTH: Parathyroid hormone
SD: Standard deviation
TIN: Tubulointerstitial nephritis
uPCR: Urine protein creatinine ratio
